# Bacteriophage Technology and Modern Medicine

**DOI:** 10.3390/antibiotics10080999

**Published:** 2021-08-18

**Authors:** Aa Haeruman Azam, Xin-Ee Tan, Srivani Veeranarayanan, Kotaro Kiga, Longzhu Cui

**Affiliations:** Division of Bacteriology, Department of Infection and Immunity, School of Medicine, Jichi Medical University, Shimotsuke-shi 329-0498, Japan; azamkla12@jichi.ac.jp (A.H.A.); xinee@jichi.ac.jp (X.-E.T.); srivani@jichi.ac.jp (S.V.); k-kiga@jichi.ac.jp (K.K.)

**Keywords:** bacteriophage, phage therapy, antimicrobial-resistant (AMR) bacteria, CRISPR-Cas13, phage engineering, modern medicine

## Abstract

The bacteriophage (or phage for short) has been used as an antibacterial agent for over a century but was abandoned in most countries after the discovery and broad use of antibiotics. The worldwide emergence and high prevalence of antimicrobial-resistant (AMR) bacteria have led to a revival of interest in the long-forgotten antibacterial therapy with phages (phage therapy) as an alternative approach to combatting AMR bacteria. The rapid progress recently made in molecular biology and genetic engineering has accelerated the generation of phage-related products with superior therapeutic potentials against bacterial infection. Nowadays, phage-based technology has been developed for many purposes, including those beyond the framework of antibacterial treatment, such as to suppress viruses by phages, gene therapy, vaccine development, etc. Here, we highlighted the current progress in phage engineering technology and its application in modern medicine.

## 1. Brief Story of Bacteriophage as Medicines

Bacteriophages (or phages for short), bacteria-infecting viruses, are the most abundant and ubiquitous organisms on earth, with a significant role in microbial population dynamics and evolutions [1]. The first documented observation that could be interpreted as phage activity dated back to 1896, when Ernest Hankin reported that the water from the rivers Ganges and Jamuna in India possessed antibacterial activity against *vibrio cholera*, suggesting the presence of a certain unidentified substance that limited the spread of cholera epidemics [2]. Similarly, two years later, Russian bacteriologist Gamaleya noted a similar antibacterial phenomenon while working with *bacillus subtilis* [2,3]. In 1910, Felix d’Herelle observed clear circular zones in bacterial lawns, for which he coined the term “plaques.” He further found that these clear zones were caused by viruses that parasitize bacteria and named them “bacterium eaters” (bacteriophages) [4]. Before the dawn of antibiotics, phages had been used to treat a wide variety of bacterial infection diseases, including cholera, dysentery, typhoid fever, skin and surgical site infections, peritonitis, septicemia, and external otitis [5,6,7]. However, the effectiveness of phage therapy has been inconsistent, which could be attributed to the lack of controls in treatment studies, less efficient therapeutic outcome, and issues pertaining to the purity of phage lysates [8]. The discovery of penicillin in the 1940s kick-started the golden age of antibiotics, which spanned over 40 years with more than 40 antibiotics developed for clinical use [9], that lead to the near-complete cessation of phage therapy in most countries.

Nonetheless, phage therapy steadily flourished in Eastern European countries, such as Russia, Georgia, and Poland. The Eliava Institute of Bacteriophage, Microbiology, and Virology (co-founded by Giorgi Eliava and Felix d’Herelle in 1923) in Georgia is one of the longest-running institutions where phage therapy has been provided to frequent bacterial diseases related to urology, gynecology, internal medicine, and pediatrics. More than 95% of patients exhibited significant improvement and recovery after phage therapy with nil adverse effects [10]. Recently, phage therapy has been re-employed using the compassionate use rationale in Europe and the United States, especially when AMR-bacteria-infected patients are without effective treatment options or are terminally ill [9]. In 2016, Tom Patterson of the University of California acquired multidrug-resistant *Acinetobacter baumanii* infection that made him comatose. He received effective and successful intravenous phage therapy on compassionate grounds to treat his condition. His recovery after phage therapy was scientifically exciting and is considered as the first successful case of phage therapy in the United States as he made full recovery from the AMR bacterial infection [11]. The positive therapeutic outcome indicated the potential of phage therapy as a last line of defense against AMR bacterial infections. To further advance such new treatments against AMR, especially focusing on phage therapy, the Interdisciplinary Center for Innovative Phage Applications and Therapeutics (IPATH) was launched at the University of California, San Diego, in June 2018 (https://health.ucsd.edu/news/releases/Pages/2018-06-21-turning-a-phage.aspx, accessed on 2 February 2021).

## 2. The Emergence of Antimicrobial-Resistant Bacteria and Phage Therapy

The emergence of antimicrobial-resistant (AMR) bacteria has been observed ever since the first use of antibiotics; however, it was regarded as a minor concern as newer antibiotics were quickly developed [12]. This therefore fueled a cycle of antibiotic discovery, the overuse of which led to the concomitant appearance of resistant bacterial strains [12]. Currently, the equilibrium of this cycle is greatly disturbed and more evident spread of AMR bacteria is being observed; yet, there is no new novel antibiotic discovery to counter these new AMR strains. With no novel discovery, most of the present antibiotics are either modified or combined versions of previously known compounds [9,13]. This interminable acquisition of antibiotic resistance could result in the development of multidrug-resistant, extensive-drug-resistant, and pan-drug-resistant strains that could pose deadly concerns due to the unavailability of antibiotic treatment choices [14]. A group of bacterial species commonly known as—*Enterococcus faecium, Staphylococcus aureus, Klebsiella pneumoniae, Acinetobacter baumannii, Pseudomonas aeruginosa,* and *Enterobacter* spp—(ESKAPE) has caused significant concern as they frequently cause severe healthcare-associated multidrug-resistant (MDR) infections and are able to escape bactericidal activity of antibiotics through multiple mechanisms [15]. It is estimated that this rise in antimicrobial resistance, if untreated, would kill 10 million people annually by the year 2050 (outnumbering the death toll attributed to cancer) and cost the global economy about 100 trillion USD, thereby necessitating the immediate development of alternative treatment strategies [16].

Recently, phage therapy has attracted renewed interest as a potential therapeutic option for treating AMR bacterial infections. The interest is evident from the increased number of PubMed-covered articles published on phage therapy (keywords: phage therapy clinical; article type: clinical trial, evaluation study, journal article, randomized controlled trial) from 2016 to 2020. About 600 articles were published in this period, whereas about 285 articles were published in 2010 to 2015. It is to be noted that these numbers in the past decade are a great leap from that of 20 years ago (1995–2000), when only 45 articles were published. This increase in article number in the past decade shows that the interest of the scientific community is switching back to phage therapy, coinciding with the increasing use of AMR in hospital settings.

Establishment of phage infection in bacteria gets initiated by the injection of the viral genome into the bacterial cell post-recognition of their specific host. Phages hijack the host bacterial machinery in favor of their two types of life cycles: lysogenic or lytic infections. In the lysogenic cycle, the phage genome gets integrated into the bacterial chromosome and continues residing as a prophage, whereas in the lytic cycle, phage particles are produced within the bacterial host cell and mature phages are finally released with the help of holin and endolysin enzymes. In nature, some phages have only a lytic life cycle (called virulent phages), while others can undergo both lytic and lysogenic cycles (called lysogenic/temperate phages). Due to their ability to lyse bacteria, phages have been adopted as therapeutics shortly after their first discovery, and phage therapy has come up as a major potential method for the treatment of AMR bacterial infections.

## 3. The Rise of Phage Engineering Technologies toward Clinical Applications

Despite the promising therapeutic potential of phages, several obstacles with regard to implementing phage therapy into clinical practice are yet to be addressed, such as (1) the emergence of phage-resistant bacteria, (2) phages with a narrow host range, (3) poor stability of phages in the blood circulation due to rapid clearance by the reticuloendothelial system (RES), (4) safety, and (5) difficulty in commercialization. Thanks to the advancement of molecular biology, phage properties can be desirably augmented through the current synthetic biology technique to overcome many of the above-mentioned shortcomings (Table 1). Limitations of phages as therapeutic agents and how phage engineering technologies resolve these issues will be addressed in detail in this section.

### 3.1. Phage Engineering for a Safer Phage Product

Some phages carry antimicrobial resistance [34], toxin [35], or virulence [36] genes in their genomes [37,38,39] and are therefore known to contribute to phenotypic changes of the host bacteria through horizontal gene transfer during viral infection, which, in some cases, is capable of enhancing their hosts’ virulence. This poses as a critical roadblock in commercializing phages as therapeutics, thus making the clinical use of phages more challenging. In addition, many genes in the phage genome encode unknown proteins that are yet to be functionally characterized; less than 50% of phage genes in most of the currently available phage genomes can actually be assigned to known proteins [39,40]. These features together limit the safety of phage therapy, due to the constraints in achieving standardization as well as to the possibility of unpredictable side effects. However, genetic engineering techniques have made possible the generation of much safer phage products. By using modern technology, the modification of lysogenic phages is strictly lytic [20] and the generation of non-propagating phages or phages lacking undesired genes is now possible. In 2019, the first successful therapeutic application of engineered phages with enhanced bactericidal activity in a patient with disseminated drug-resistant *Mycobacterium abscessus* was reported [41]. These scientific and technical advancements aid the preparation of diverse types of desired engineered phages, which are pivotal in future modern medicine.

### 3.2. Phage Engineering to Broaden the Host Range and Limit the Emergence of Phage-Resistant Bacteria

Rapid emergence of phage-resistant bacteria is often observed during in vitro experiments [18,42,43,44,45]. Though it was speculated that this phenomenon is unlikely to occur during phage therapy, a few recent studies have demonstrated that the emergence of phage-resistant bacteria during the course of treatment is frequent and almost inevitable [9,37,42,46,47]. The use of phage cocktails that target different bacterial receptors and combined treatment with phages and antibiotics have been suggested to hinder the development of phage resistance, and at the same time, the host range of an individual phage or a mixture of phages can be expanded [8,9,11,48]. However, the isolation and characterization of the constituent phages is tedious and requires strenuous regulatory approval for their therapeutic application. To circumvent these issues, the phage host range can be expanded through modification of the phage tail ligand protein [19,49] or key determinants of the phage–host interaction, either by homologous recombination with closely related phages or by rebooting the synthesized genome. Synthetic biology techniques allow the generation of various chimera phages in the T2, T4, and T7 families, each of which targets different bacterial receptors for synergistic therapy and thus delay the emergence of phage-resistant clones [50]. Viable customized *L. monocytogenes* phages with an expanded host range have also been generated using a similar approach [22].

### 3.3. Phage Engineering for Stabilizing Phages in Blood Circulation

Alteration of the viral capsid amino acid [27,51] and conjugation of PEG onto phage particles (PEGylation) are the present techniques proposed to improve phage stability in the blood circulation [28]. Mutation in the major capsid (E) protein of the lambda phage was sufficient to boost phage stability in the blood circulation by up to 16,000-fold compared with the wild type [27]. The PEGylation technique, in contrast, was inspired by previous reports demonstrating enhancement of the therapeutic potency of protein drugs [52,53,54] as well as a reduction in humoral and cell-mediated responses of humans against mammalian viruses [55,56] when the PEG molecule is covalently bound to those drugs/viruses. PEGylated *Listeria* phage A511 and *Salmonella* phage Felix O1 elicited significantly reduced levels of IFN-γ and IL-6 in naive and immunized mice [28]. Twenty-four hours post-injection, improved stability in the blood circulation (up to 100 times) was observed in *Listeria* phage A511, whereas Felix O1 showed no significant improvement [28].

### 3.4. Phage Engineering for Phages That Can Be Easily Commercialized

Due to the presence of many unknown genes in the phage genome, scientists are yet to tackle the safety concern of poorly characterized phages. Therefore, instead of using natural propagating phages, phage derivatives, such as phage endolysin, are being investigated as alternative therapeutic options to natural phages. Phage derivatives are generally considered to be safer than natural phages and hence can be passed through regulatory approval smoothly. Endolysin has been widely used for therapeutic application and has even passed phase I/II clinical trials in some countries [9,29]. However, application of endolysin against Gram-negative bacteria is still challenging due to the presence of a bacterial outer membrane layer that shields the cell wall’s peptidoglycan from being accessed and damaged by the enzyme, thereby hampering the anticipated bacterial killing. Nonetheless, replacing certain endolysin amino acids with hydrophobic ones [57] or fusion with membrane-destabilizing peptides (artilysins) [58] is shown to overcome this problem effectively.

Beside phage-derived products, phage particles encapsulating genetic elements other than the phage’s own genome have recently been explored. A phagemid is a type of plasmid containing a phage origin of replication (ORI), including phage-packaging sites [59]. Due to the presence of genetic elements that signal for packaging, a phagemid can be packaged into a phage capsid, generating non-infectious daughter phage particles that carry phagemid DNA. These phagemids transport well-characterized known genes and are advantageous owing to their inability to replicate; additionally, new functional foreign genes can also be added onto the phagemid. Alternatively, phage-inducible chromosomal islands (PICIs), a recently discovered family of pathogenicity islands, can also be packaged into a phage capsid. PICIs mobilize among bacterial species at high frequencies (horizontal gene transfer), representing a potential tool for synthetic gene delivery. These elements are located in the bacterial chromosome and have the ability to interfere with phage reproduction by hijacking the phage machinery to package their own [60]. Upon infection by a phage (helper phage) or SOS induction of a temperate phage, PICI elements excise, replicate, and are efficiently packaged into a phage capsid at the expense of the phage packaging machinery by ceasing phage propagation [60,61]. The best-studied PICIs are the *S. aureus* pathogenicity islands (SaPIs) [62,63]. A previous study has shown that replacement of a SaPI’s toxin genes with antibacterial cargoes could facilitate the generation of antibacterial drones that target the causative bacteria in an animal model of infection [31].

In addition to the aforementioned approaches, in situ expression of external proteins (phage arming) can not only enhance phage antimicrobial activity but also reprogram the phages to meet the demand for functional phage therapy. Phage arming has been adapted to generate functional phages that can decompose bacterial biofilm or eradicate capsule-producing bacteria by introducing genes encoding biofilm depolymerases [64] and capsule depolymerases [65], respectively, into a phage capsid. Similarly, phages have been exploited to deliver small regulatory RNAs for silencing of antibiotic resistance determinants [23] and to carry genes that encode proteins capable of increasing the susceptibility of bacteria to antibiotics [25].

## 4. Development of Gene-Specific Antimicrobials

### 4.1. The Use of a Clustered Regularly Interspaced Short Palindromic Repeat (CRISPR)-Cas System as a Gene-Specific Antimicrobial

To stand up to the increasing threats of AMR bacteria, our group sought to develop a synthetic phage that can specifically target and kill such bacteria. We used the CRISPR-Cas system, a well-known, revolutionary gene-editing tool that edits genes of interest by employing CRISPR RNA (crRNA) to guide the endonuclease and Cas protein to cleave the target nucleic acid.

In March 2011, a protein–nucleic acid complex consisting of type II-A effector Cas9 (an enzyme that cleaves double-stranded DNA) and guide RNA was discovered to be a prokaryotic adaptive immune system responsible for protecting bacteria from foreign genetic elements, such as plasmids and phages [66]. Following its discovery, studies on genome editing using Cas9 have been advancing all over the world. Programmable removal of bacterial strains using genome-targeting CRISPR-Cas9 was reported in 2014 [67]. In the same year, a gene-targeted antibacterial agent capable of targeting antibiotic resistance and/or virulence genes of *E. coli* [68,69] and *S aureus* [70] was generated using non-replicative phagemids. Other than Cas9, Cas3 (type I-E) having the same DNA-targeting characteristic as Cas9 is under development. The protein–nucleic acid complex containing Cas3 is known to degrade the surrounding single-stranded DNA after cleaving the target DNA; thus DNA repair is unlikely to occur, as seen with Cas9 editing [71]. In 2015 and 2017, programmed temperate phages loaded with Cas9 or Cas3 to target *E. coli* and *S. aureus*, respectively, blocked the transmission of drug resistance genes effectively [71,72]; in 2018, the therapeutic effect of Cas9-loaded phages was reported in a mouse infection model [31]. In 2019, Johnson & Johnson of the United States invested a huge amount of funds in Locus Bioscience to evaluate the efficacy of Cas3-loaded phages [73] for the treatment of respiratory and other organ infections.

Cas9 and Cas3 are both nucleases that target DNA; when such DNA-cleaving nucleases are employed for antibacterial treatment, there is an unexpected risk of genetic variation after nuclease editing in the target gene, owing to the DNA repair mechanism of bacteria. In addition, if the target genes are located on bacterial plasmids (that are non-essential for bacterial survival), targeted editing using Cas9 or Cas3 will not result in eradication of bacterial cells [67]. It is to be noted that many of the clinically important antibiotic resistance genes are present on plasmids [74]. Therefore, to tackle this problem, our group proposed to use another class of the CRISPR-Cas system, CRISPR-Cas13a [24,75]. Cas13a is an RNA-targeted nuclease whose function was identified in 2016 [75]. The unique feature of Cas13a is that this nuclease undergoes structural changes after recognizing the target gene [76], resulting in indiscriminate degradation of the host bacterial RNAs and subsequent suppression of bacterial growth. We exploited CRISPR-Cas13a for the development of a gene-specific bactericidal agent that has the potential to be applied for various purposes. Our study revealed that Cas13a, after recognizing the target RNA inside the host cells, exhibits its action by not only suppressing the growth of host bacteria but also killing them [24,77].

### 4.2. CRISPR-Cas13a-Based Antibacterial Nucleocapsid

**Figure 1:**
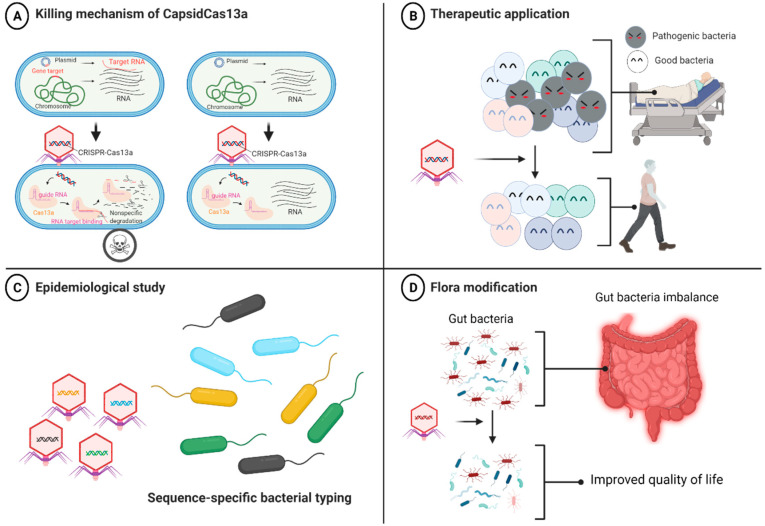
Mechanism of action of antibacterials equipped with CRISPR-Cas13a (CapsidCas13a) and its potential applications. (**A**) Selective killing activity of CapsidCas13a. CRISPR-Cas13a is injected into the bacterial cell and translated into guide RNA (crRNA) and Cas13a proteins. As crRNA binds to the target RNA sequence, Cas13a protein changes into its active form that collaterally cleaves any nearby RNAs, causing host cell death. (**B**) Potential therapeutic application of CapsidCas13a for selective killing of pathogenic bacteria population. (**C**) CapsidCas13a bacterial typing method can be used as an epidemiological tool to identify variation in the sequence of genes, thereby discriminating different bacterial isolates within the same species (genotyping). (**D**) Flora modification to improve the quality of life [79].

We then developed a series of CRISPR-Cas13a-based antibacterial nucleocapsids (CapsidCas13a) by packaging programmed CRISPR-Cas13a into a carrier phage capsid using the PICI packaging system for *E. coli* [60] and the SaPI packaging system for *S. aureus* [62,78]. We observed that CapsidCas13a sequence-specifically killed *E. coli* carrying various carbapenem resistance genes (*bla*IMP-1, *bla*OXA-48, and *bla*VIM-2) as well as MRSA (carrying the methicillin resistance gene *mecA*) by targeting the AMR genes regardless of their location, either on the chromosome or on the plasmid [24]. Moreover, it also precisely killed *E. coli* by targeting toxin-encoding genes (*stx1 and stx2*). By using a *Galleria mellonella* infection model, we confirmed that CapsidCas13a significantly improves host survival compared to the control, indicating its potential as an antibacterial agent [24]. CRISPR-Cas9-based antibacterial nucleocapsids (CapsidCas9) were also constructed in one of our previous studies for comparison with CapsidCas13a. We observed that CapsidCas9 could only kill bacteria by targeting genes located on the chromosome but not on the plasmid, reflecting the inability to kill the bacteria if the target gene is on the plasmid. This is because Cas9-mediated plasmid DNA cleavage is not deleterious for bacteria, whereas Cas13a induces cell death through collateral non-specific cleavage of RNA, ensuing target RNA recognition (Figure 1A). We concluded that our system has at least three major applications (Figure 1B–D): (1) as an antibacterial agent targeting any bacterial genes, including antibiotic resistance, toxin, and virulence genes; (2) for the editing of bacterial flora by targeting and eliminating a specific bacterial population, while maintaining other irrelevant bacterial populations; and (3) as a simple and inexpensive bacterial gene detection tool for bacterial identification and molecular epidemiological investigation without the need for amplification of nucleic acids or optical devices (e.g., we could simply detect bacterial lysis after addition of phagemids). Since the synthesized CapsidCas13a does not carry the phage genome, it is safer than the natural phage and should be relatively easier to be put into practical use as a therapeutic drug.

## 5. Other Applications of Phages

Phage engineering has also been used for developing vaccines recently. Phage display [80] is the core technique that is used for phage-based vaccine design for antigen expression [81]. Principally, the nucleotide sequence of a vaccine or an antigen is cloned into the phage DNA encoding the capsid protein at a specific location (either a major capsid or an accessory protein). The expression of an antigen on the phage surface is picked up by the immune system to render an immune response against the antigen. In the case of DNA vaccines, a gene expression cassette containing an antigen sequence can be introduced into the phage genome. The phage is then tailored to carry ligand proteins that target antigen-presenting cells (APCs). Upon administration of such a chimera phage, APCs recognize and take up the phage, releasing the genomic DNA, which could later lead to expression of the antigen. Recent studies have reported that oral immunization with MS2 virus-like particles (VLPs) that express human papillomavirus (HPV) protein could protect mice against oral and genital HPV infection [82]. In another study, melanoma neoantigens expressing phage T7 elicited an anti-melanoma immune response upon in vivo administration [83]. This field is ever-expanding now, and more phage-based vaccines are expected to be researched, especially in regard to COVID-19, where a phage-based vaccine is being developed now [84].

Another emerging application of phage engineering is the development of phage-based biomaterials for tissue regeneration [85]. The idea was inspired by the fact that phages are human-safe bacterial viruses that can be used as suitable nanoscaffolds, especially filamentous phages due to their unique nanofiber-like morphology. They can be synthesized in an error-free format, self-assemble into an ordered scaffold, display multiple signaling peptides, and serve as a platform to screen novel signaling or homing peptides. In addition, by generating phage libraries expressing a wide array of peptides, by a technique known as biopanning, scientists have been able to successfully screen many peptides that specifically bind to selective targets, including cells [86], tissues [87,88], polymers [89], proteins [90], and inorganic crystals [91]. This technique is particularly interesting when a peptide-displaying phage is used for tissue regeneration [85]. The future direction of this field includes (1) development of an engineered phage-based substrate for controlling the fate of induced pluripotent stem cells (iPSCs), considering the unique advantage of using iPSCs in tissue regeneration, and (2) realization of in vivo tissue regeneration studies by using in vivo biopanning.

## 6. Future Direction

AMR bacteria have become a major public health challenge worldwide. This crisis along with no newer antibiotics has revived the interest in phage therapy. In the era of synthetic biology, the major difference from conventional phage therapy is the use of artificially engineered phages with enhanced therapeutic properties. The engineered phage can be developed not only for antibacterial treatment, but also for the detection of microorganisms and as a biomaterial [30,85]. Phages can also carry long-chain DNA or display proteins and can function as an ideal delivery system [81,92], substantiating their potential to be used in vaccine development, gene therapy, and virus suppression.

Phage synthesis technologies have made great strides in the past 5 years. Rebooting of an exogenously synthesized phage genome has become possible and efficient in both Gram-positive [20] and Gram-negative [19] bacteria. In addition, a cell-free synthesis method cell-free transcription–translation (TXTL) [32] has achieved great progress recently. We believe that in the near future, preparing customizable phages will be as easy as synthesizing oligonucleotides. In that case, the use of phages as a substitute for antibacterial drugs will become a reality. In addition, a synthetic approach is expected to be used to unveil the biological properties of phages that have not been well elucidated. Although the analysis of model phages, such as the so-called T series, λ, MS2, Qβ, ΦX174, Mu, Φ29, P1, P2, and P22, has made significant progress to some extent [93,94,95,96,97,98,99,100,101,102], considering a massive amount of phages in nature, which is about 10^31^, many are still awaiting discovery. Moreover, Jumbo phages, which have large genome sizes, have been discovered, but most of their genetic properties remain unknown [103,104,105]. A recent study discovered the presence of CRISPR-Casφ (the smallest CRISPR-Cas system to date) in the genome of the jumbo phage [105].

Phage engineering technology is expected to be continuously developed from now on. Highly stable and safe phage particles equipped with strong killing ability and a broad host range are likely to be used as a modern medicine in the future. The use of an auxiliary component, such as the CRISPR-Cas system, to improve the phage therapeutic potential is an epoch-making approach and has been shown to be useful for various applications, which were not possible to be achieved using existing antimicrobial agents. Non-propagating and genome-free features make the approach more suitable for therapeutic applications. Before the establishment of clinical phage therapy, we direct our study toward (1) optimization of the loaded gene(s) and phage host range, (2) stabilization of phage preparation, and (3) evaluation of phage purification and administration methods. We suggest that phage applications can extend beyond medical field into many other sectors, such as veterinary medicine, agriculture, forestry and fisheries, natural environment conservation, and food manufacturing [106].

## Figures and Tables

**Table 1:** Obstacles in phage therapy and emerging approaches in synthetic biology.

	Obstacle in Phage Therapy	Conventional Approach	Synthetic Approach
1	Narrow host range	Application of phage cocktail [17,18].	Genetic manipulation of receptor-binding protein [19,20].
2	Emergence of phage-resistant bacteria	Phage cocktail; combination of antibiotic and phage [9,21].	Genetic manipulation of receptor-binding protein [22]; incorporation of small RNAs or CRISPR-Cas system to silence antibiotic resistance determinant [23,24] or delivery of genes encoding proteins capable of increasing bacteria susceptibility to antibiotics [25].
3	Low stability of phage in blood circulation due to rapid clearance by reticuloendothelial system (RES)	Multiple doses of phage administration [26].	Introduction of mutation in phage capsid protein [27]; introduction of PEG into phage particle (PEGylation) [28].
4	Safety concern due to difficulty of standardization and the presence of many unknown genes in phage genome	Application of phage-derived endolysin [29].	Development of well-characterized, non-propagating phages [30]; development of antimicrobial payload using a phagemid and phage-inducible chromosomal islands (PICIs) [24,31].
5	Presence of potential hazardous genes (toxin, virulence, and antibiotic resistance genes) in phage genome	Only strictly virulent phage is recommended for therapy [9], and whole-genome analysis should be done in the first place.	Custom-made phage can be generated easily using current techniques [19,20,32].
6	Safety concern due to low purity of phage preparation and potential toxin contamination from bacterial propagation cell	Removal of toxins by CsCl purification and ion exchange column [9] or affinity chromatography [33].	Phage production using cell-free system, such as cell-free transcription–translation (TXTL) [32].

## Data Availability

The data presented in this study are available on request from the corresponding author.
